# Coherent Longitudinal Acoustic Phonon Approaching THz Frequency in Multilayer Molybdenum Disulphide

**DOI:** 10.1038/srep05722

**Published:** 2014-07-17

**Authors:** Shaofeng Ge, Xuefeng Liu, Xiaofen Qiao, Qinsheng Wang, Zhen Xu, Jun Qiu, Ping-Heng Tan, Jimin Zhao, Dong Sun

**Affiliations:** 1 International Center for Quantum Materials, School of Physics, Peking University, Beijing 100871, P. R. China; 2 Collaborative Innovation Center of Quantum Matter, Beijing 100871, P. R. China; 3 State Key Laboratory of Superlattices and Microstructures, Institute of Semiconductors, Chinese Academy of Sciences, Beijing, 100083, P. R. China; 4 Beijing National Laboratory for Condensed Matter Physics and Institute of Physics, Chinese Academy of Sciences, Beijing 100190, P. R. China

## Abstract

Coherent longitudinal acoustic phonon is generated and detected in multilayer Molybdenum Disulphide (MoS_2_) with number of layers ranging from 10 to over 1300 by femtosecond laser pulse. For thin MoS_2_, the excited phonon frequency exhibits a standing wave nature and shows linear dependence on the sample thickness. The frequency varies from 40 GHz to 0.2 THz (10 layers), which promises possible application in THz frequency mechanical resonators. This linear thickness dependence gradually disappears in thicker samples above about 150 layers, and the oscillation period shows linear dependence on the probe wavelength. From both the oscillation period of the coherent phonon and the delay time of acoustic echo, we can deduce a consistent sound velocity of 7.11*10^3^ m/s in MoS_2_. The generation mechanisms of the coherent acoustic phonon are also discussed through pump power dependent measurement.

Two dimensional (2D) materials down to thickness of single or few layers attract interests for their associated unique properties in electronics and optoelectronics, and recently developed valleytronics^1^,^2^,^3^,^4^,^5^. As a representative 2D material beyond graphene, molybdenum disulphide (MoS_2_) got considerable attention because of its unique electric and optical properties^5^,^6^,^7^,^8^,^9^,^10^,^11^,^12^, which makes it especially interesting and promising for possible future electronics^13^, optoelectronics^14^ and valleytronics applications^4^,^5^. Among many device physics toward future applications in these directions, it's not only crucial to understand the behavior of carriers, but also vital to understand the environmental surroundings of the carriers: the lattice or phonon, and especially its interaction with electron, which plays a central role in carrier mobility^15^ and device heat dissipation^16^.

In terms of studying vibrational properties of materials, Raman spectroscopy has been very successful in studying high frequency optical phonons and their couplings to electrons, especially in 2D crystals such as graphene and MoS_2_ in few-layer regime^17^,^18^,^19^. However, for low frequency optical phonon and acoustic phonon, the low phonon energy renders it difficult to be observed by conventional Raman spectroscopy^20^. In this paper, using femtosecond pump-probe technique in reflection geometry, we are able to generate and detect the coherent acoustic phonon oscillation in MoS_2_ from thick flakes (above 1000 layers) down to 10 layers in time domain. The coherent phonon oscillation is identified to be out of plane coherent longitudinal acoustic phonon (CLAP) mode from layer dependent and wavelength dependent measurement of oscillation period^21^,^22^. Interestingly, we observe that the oscillation period increases linearly with number of layers due to the formation of a standing sound wave in the thin film and shows no clear dependence on probe wavelength. In contrast, when the sample thickness increases, the oscillation period evolves to be independent of thickness but linearly dependent on probe wavelength. Further pump power dependent studies indicate the major mechanism for the coherent phonon generation is coherent Brillouin scattering under relative low pump excitations.

Coherent collective excitations of phonons, magnons, charge density wave excitations, etc, which carry definite phase and temporal information, have so far been generated and detected using ultrafast spectroscopy^23^,^24^,^25^,^26^,^27^,^28^,^29^. In either transient differential transmission (ΔT/T) or differential reflection (ΔR/R) geometry of the pump-probe setup, the pump induced ΔT (ΔR) signal oscillating at phonon frequencies is detected as a function of delay time relative to the pump pulse. The mechanisms of coherent phonon generation are well studied in the literatures^24^,^30^,^31^,^32^: for optical phonons in transparent media, it's mainly initiated via impulsive stimulated Raman scattering (ISRS)^33^,^34^; in absorbing materials, mechanisms can be either displacive excitation of coherent phonons (DECP)^35^,^36^ and resonant ISRS^24^. For coherent acoustic phonon generation, the acoustic counterpart to ISRS is impulsive stimulated Brioullion scattering (ISBS) which gives rise to sound through photoelastic modulation of refractive index. This mechanism usually shows linear pump power dependent in transparent media^37^; in absorbing substances, similar to the optical phonon case, light may be coupled to sound directly through the photoelastic mechanism or indirectly through laser induced thermomodulation^38^: the pump pulse gives a sudden temperature rise which converts into strain because of thermal expansion. Figure 1 illustrates a scheme of CLAP generation and detection in MoS_2_: the ultrafast pump pulse is absorbed at the top layers of the MoS_2_ sample, the absorbed photon energy gives rise to a transient phonon temperature increase within the illuminated area, which then sets up a transient stress. The stress induces a strain wave (coherent longitudinal acoustic phonons), which propagates away from the sample surface at the speed of the longitudinal acoustic phonons. This CLAP wave modifies the local dielectric constants, and when the probe pulse is incident onto the sample, the reflection of the probe light is modified by the local dielectric constant modification due to the generation of CLAP, thus the periodic CLAP oscillations can be detected.

## Methods

**Figure 1:**
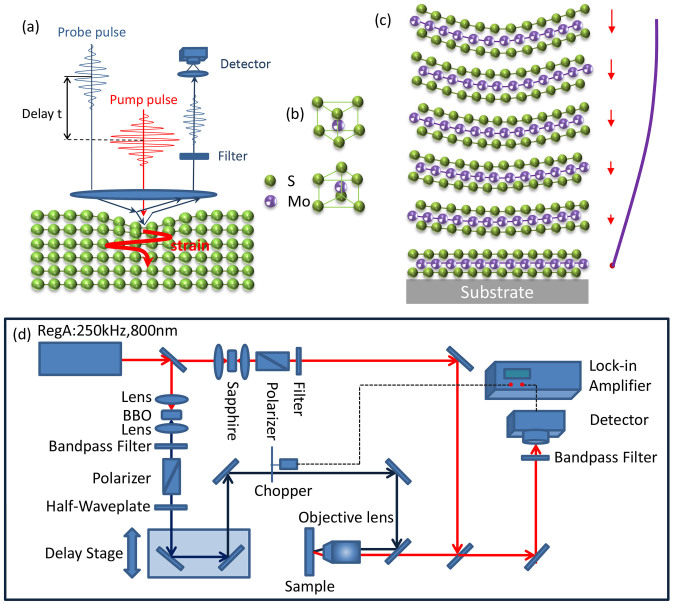
Schematic diagram and experimental setup of coherent phonon oscillation in few layer and bulk MoS_2_ (a) Schematic diagram of generation and detection of the longitude acoustic coherent phonons in the MoS_2 _crystal. (b) The crystal structure of MoS_2_. It contains two unit cells of MoS_2_ monolayer in the diagram. (c) Schematic diagram of the confined mode in the thin layers MoS_2_ which is standing waves of in phase atomic oscillations. As the case in this figure, one surface of the film is free (zero-stress boundary condition), and the other is in contact with the substrate, which gives an approximately zero-displacement boundary condition. (d) Schematic diagram of experimental setup.

Our experimental setup is shown in Figure 1(d), a mode-locked Ti: Sapphire laser generates 60 fs pulses with central wavelength of 800 nm and 250 kHz repetition rate. A small portion of this beam is reflected by a beamsplitter and then focused to a beta barium borate (BBO) crystal to generate second harmonic at 400 nm and used as a pump pulse unless in a pump wavelength dependent measurement. The majority of the 800 nm beam is focused on a 2 mm sapphire plate to generate white light super continuum. Then a narrow band-pass filter is used to filter the desired probe wavelengths for the pump-probe measurement. The pump pulses pass through a variable optical delay line to control the delay time between the pump and probe pulses. Both pump and probe pulses are focused through a 40X near-infrared objective onto the sample which is placed in a liquid-nitrogen-cooled cryostat. The reflected probe is collected by the same objective lens and detected by a Si photodetector and lock-in amplifier referenced to 5.7 kHz mechanically chopped pump. A narrow bandpass filter at probe wavelength is used to prevent the detection of reflected pump before the detector. The probe spot size is estimated to be below 2 μm while the pump spot size is slightly larger. Typical pump and probe power used are 20 μW and 10 μW respectively unless in a pump power dependent measurement. Most data presented in this paper are measured with 400-nm pump and 880-nm probe at room temperature unless specified.

Multilayer MoS_2_ flakes are fabricated by standard mechanical exfoliation from natural bulk MoS_2_ crystals^39^ on a Si/285 nm SiO_2_ substrate. The bulk MoS_2_, as an indirect-gap semiconductor with a band gap of 1.29 eV, is built up of van der Waals bonded S-Mo-S units. Each of these stable units (referred to as a MoS_2_ monolayer) consists of two hexagonal planes of S atoms and an intermediate hexagonal plane of Mo atoms coordinated through ionic-covalent interactions with the S atoms in a trigonal prismatic arrangement^8^ (Figure 1b). The thickness of each layer (one Mo layer sandwiched by two S layers) is 0.65 nm. We can identify the thickness of few layer flakes (monolayer to 5 layers) by optical contrast in optical microscope and then confirm the results by the frequency difference between the A_1g_ and E^1^_2g_ peaks in Raman spectroscopy^18^. The thickness of relative thicker sample (from 6 L to 20 L) is initially identified by atomic force microscopy (AFM) working at the tapping mode with 0.1 nm resolution in vertical direction and 8 ~ 10 nm resolution in transversal direction. To avoid the error from the interaction of MoS_2_ and the substrate, we further confirm the AFM results by measuring the interlayer shear mode in a low wavenumber Raman spectroscopy^40^ (see supporting information). For the samples beyond 20 layers, AFM is solely used to measure the total thickness.

## Results and Discussion

**Figure 2:**
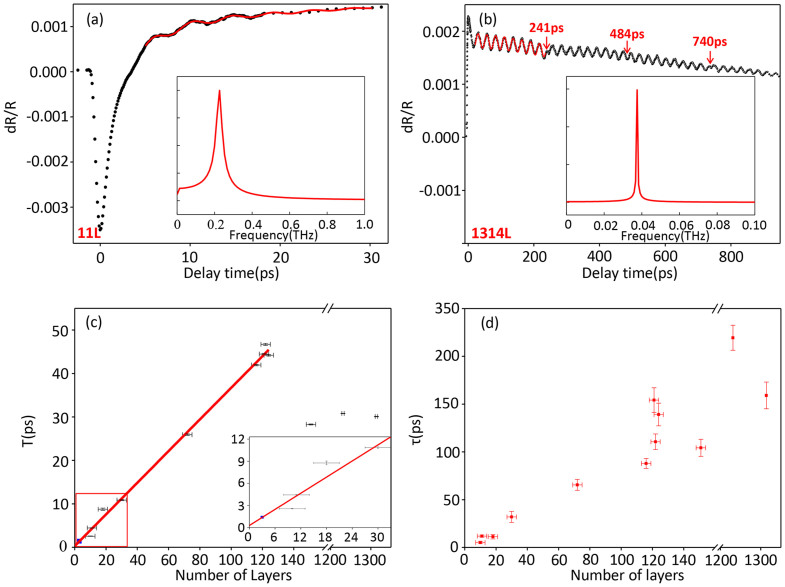
The layer number dependence of coherent phonon oscillation periods. (a) Transient reflection spectra (ΔR/R) of the 11 L MoS_2_ film with pump wavelength at 400 nm and probe pulse wavelength at 880 nm. The inset shows the fast fourier transform (FFT) of the oscillation. (b)Transient reflection spectra (ΔR/R) for 1314 L MoS_2_ film with pump wavelength at 400 nm and the probe pulse wavelength at 800 nm. The inset shows the FFT of the oscillation. (c) Oscillation period as a function of number of layers with pump wavelength of 400 nm and probe wavelength of 880 nm. The red line is a linear fit for up to 122 layers. The blue data point is taken from Ref.^45^. The inset zooms in the area marked by red rectangular. (d) Damping constant as a function of layer number.

Figures 2 (a) and (b) illustrate the representative full scan of transient reflection spectra of the MoS_2_ films with 11 layers (11 L) and 1314 layers (1314 L), respectively. In both cases, the responses consist of two components: one is a step like jump at time zero followed by decay (and zero crossings in certain thicknesses) due to the relaxation and recombination dynamics of excited carriers^41^,^42^,^43^,^44^,^45^; the other is a damped oscillation, whose period shows no clear temperature and pump probe polarization dependence (see supporting information). The carrier dynamics in monolayer MoS_2_ is currently a subject of intense interest, numerous ultrafast spectroscopy works have been performed to study it^44^,^45^,^46^,^47^,^48^,^49^. Recently work indicates many body effect may dominate the pump probe response in monolayers^47^. For multilayer samples, the transient reflection response also evolves due to the band structure evolution as sample thickness increases^46^. However, this part of response is not the major focus and it doesn't affect any discussion or conclusion about CLAP that is studied. For the second component, the oscillation can be identified to be CLAP oscillation as further investigated.

The insets of Figures 2 (a) and (b) show the fast Fourier transform (FFT) of the damped oscillation and give oscillation frequency of 0.226 THz for 11 L and 0.038 THz for 1314 L. The periodic oscillation is universal and clear on all samples that we measured with thickness above 10 L, more full scans of transient reflection spectra with different number of layers are available in supporting information. Here we use the following empirical function to fit the measured transient reflection spectra^50^,^51^,^52^: 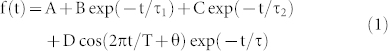
The top three parts of the function describe the background due to the recovery of the exciton population while the last exponentially decaying sinusoid part is used to describe the coherent phonon oscillation. A, B, C, D are fitting parameters and t is the delay time between pump and probe pulse. Both τ_1_ and τ_2_ are parameters relate to carrier dynamics, while T and τ are oscillation period and damping time of the oscillations.

Figure 2(c) shows the oscillation period as function of total number of layers, two different regions can be identified from the layer dependence plot: in the first region, say below 122 L, the oscillation period shows linear dependence on sample thickness. This linear dependence persists until about 122 L with oscillation period increased linearly to 46.71 ps; in the second region, as the sample becomes thicker, the linear dependence fails: for 151 L, the oscillation period drops to 28.27 ps and the periods stay around 30 ps for very thick samples (1231 L and 1314 L). Figure 2 (d) shows the damp time as function of sample thickness. The oscillations are more pronounced for thicker samples, and the damping time become longer. This is mainly because the effect from the substrate is greater in thinner samples and the oscillation gets damped each time it reaches the interface.

**Figure 3:**
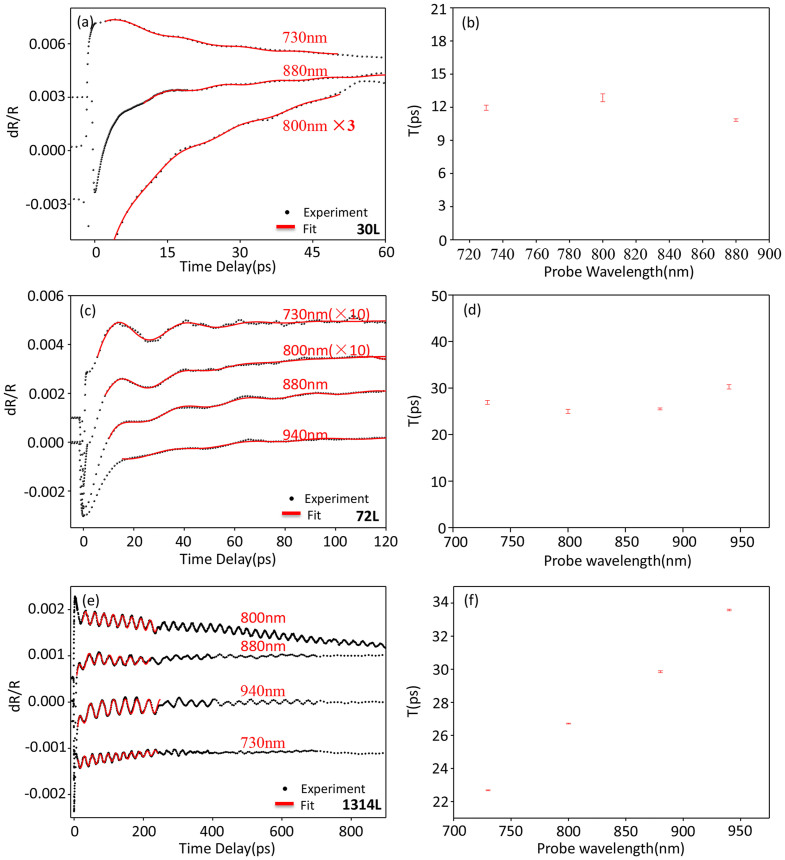
The probe wavelength dependence of coherent phonon oscillations. (a)(c)(e) Transient reflection spectra of the 30 L, 72 L and 1314 L sample with 400-nm pump and different probe wavelength (Plots have been shifted vertically for clarity). (b)(d)(f) The probe wavelength dependence of the coherent phonon oscillation period for 30 L, 72 L and 1314 L samples respectively.

The observed oscillation signals in the thin samples (<122 L) are attributed to standing wave CLAP according to its linear thickness dependence. Similar phenomenon has previously been studied in other material platforms such as As_2_Te_3_^22^, semiconductor quantum dots^53^ and nanoparticles^54^. As shown in Figure 1(c), the laser pulse generates a plane stress wave (or longitudinal acoustic phonon) which bounces back and forth in the film and form a standing wave with wavelength of the stress wave purely determined by thickness of the thin film. The strain induced by this stress wave changes the gap in the MoS_2_ and in this way changes the absorption of the time-delayed probe pulse. The situation of our studies is shown in Figure 1(c): one surface of the film is free (zero-stress boundary condition), and the other is in contact with the substrate, which gives an approximately zero-displacement boundary condition. As given in the literature^22^, the period of oscillation is: 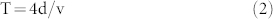
where d is the film thickness and v is the sound velocity. Based on this simple relationship, we can deduce a sound velocity of 7.11 × 10^3^ m/s from the slope of linear fit in Figure 2(c). We also note the fitting line pass through the origin which indicates the propagation of stress wave in SiO_2_, if exists, doesn't affect the oscillation period. A probe wavelength dependent study is shown in Figure 3 (b) and (d), the oscillation period doesn't show any clear dependence on wavelength for 30 L and 72 L, which matches equation (2). For samples below 10 L, no clear periodic oscillation is observed in our measurements. However we notice in the literature that similar periodic oscillation is observed down to 3 layers using similar ultrafast pump probe measurement but in a probe-transmission detection geometry. These oscillations were originally attributed to coherent optical interlayer shearing or breathing phonon mode in the literature^55^. Interestingly, we note the oscillation period of for 3 layers observed in Ref 55 is 1.4 ps, which falls on the linear fit extrapolated to 3 layers as shown in the inset of figure 2(c), so we speculate those oscillations are also due to CLAP instead of coherent optical phonon oscillations. The reason we don't observe it is probably because the coherent phonon signal is stronger in transmission geometry compared to reflection geometry used in our experiment as limited by the micrometer size of exfoliated samples. We further point out that the approaching THz frequency oscillation for thin layer MoS_2_ potentially provides a promising material platform for high frequency mechanical resonators working in challenging THz frequency range^56^. Although THz frequency acoustic phonon has been previously demonstrated in highly absorbing substances so that the photon penetration depth becomes the length scale for the phonon wave vector^57^, thin 2D material like trilayer MoS_2_ still promises a more realistic platform for THz frequency mechanical resonator applications.

At intermediate thickness, a sudden drop of oscillation period from 46.71 ps (122 L) to 28.27 ps (151 L) is observed. This indicates the conditions to form standing wave start to break down within this thickness range. This phenomenon can be understood from two aspects. First, if we assume 2.3% absorption per layer for 400 nm (actually above 5% for single layer reported in Ref. 8, but we need to take the saturate absorption for femtosecond pulse into account), the pump beam is attenuated by 94% after passing through 122 L. Within 122 L, the stress wave are partially excited by pump beam directly in each layer, and a standing wave can be formed due to the interference between forward and backward travelling stress wave. Second, if we estimate the round trip time of stress wave for 122 L, it is 22.43 ps, which is about half period of 46-ps CLAP oscillation, thus a standing wave can barely be formed from the interference of forward travelling and back travelling stress wave. While for 151-layer sample, the round trip takes 27.76 ps, which is close to the CLAP period that is measured. We also speculate hybrid modes (standing wave/travelling wave) and higher order standing wave mode can happen which further complicates the layer dependence of oscillation period in this transition region.

In the second regions, for very thick samples, the behavior of oscillation period changes dramatically. Figure 3(f) shows the oscillation period has clear linear dependence on probe wavelength in a 1314 L sample. However, the periods stay the same for different thickness as shown in Figure 2(b). This indicates the detected periodic oscillation is different from the standing wave CLAP in thin samples. In these very thick samples, standing wave cannot be formed due to the large thickness of the sample. For 1314 L sample, once the sample is excited, the photo-induced acoustic phonon wave is generated near the surface and travels into the substrate; then it takes the stress wave about 240 ps to reach the substrate and be reflected back to the surface assuming a sound velocity of 7.11*10^3^ m/s. This round trip time is far larger than the 30-ps oscillation period observed.When the back reflected stress wave reaches the sample surface, it produces the first “acoustic echo” at 240.3 ps delay as labeled by the red arrow in Figure 2(b). The position of “acoustic echo” also matches the round trip time in 1314 layers with sound velocity of 7.11*10^3^ m/s. In this case, the oscillations in the transient reflectance responses arise from interference between probe laser photons reflected from the front surface and from the traveling coherent longitudinal acoustic phonon plane wave, so the period has wavelength dependence. On the other hand, the period doesn't depend on the sample thickness, since only the top layers come into play and the layers underneath is not probed due to the probe absorption. The behavior of oscillation period is already well studied in the literature^21^, it has the form: 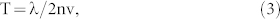
Where the λ is the probe wavelength and n is the refractive index of the sample, v is the sound velocity. Based on Equation (3), using the sound velocity of 7.11 × 10^3^ m/s, we get refractive index of 2.06 for probe wavelength of 880 nm. There are huge discrepancies of refractive index in the existing literatures^58^,^59^,^60^, it ranges from 2.79^58^ to 2.8 ~ 4 for different samples^60^, and even 6 for 700 nm^59^. The value measured from our experiment is significantly smaller than 2.79^58^, which is the lowest refractive index reported^54^. However, considering the large error bar of refractive index in the literatures and many other factors, such as stress wave induced vertical lattice displacement, lattice mismatch induced lateral lattice displacement in thin samples and probe absorption induced the refractive index change, n = 2.06 for 880 nm is deemed within reasonable range under acoustic stress.

**Figure 4:**
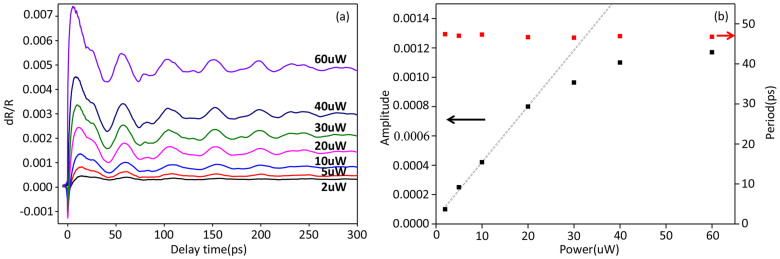
Pump power dependent of the coherent phonon oscillations. (a) Power dependent of the coherent phonon oscillation of 122 L sample. The pump wavelength is 400 nm and the probe wavelength is 880 nm while the probe power is 10 μW. (b) Power dependent of the amplitude of the oscillation.

Now we turn to investigate the generation mechanism of CLAP by impulsive optical excitation in our experiment. 400 nm pump wavelength is far above the exciton and indirect gap of MoS_2_, thus the material is absorptive to this wavelength, but the pump pulse can still penetrate through thin samples. To identify which specific CLAP generation mechanism dominates in our experiment, we performed pump power dependent studies of CLAP in 122 L sample as shown in Figure 4. Over large pump power range in 122 L, oscillation amplitude is almost linear with power in low power range and then it starts to saturate. This indicates both ISBS and thermal expansion contribute to the CLAP generation, but ISBS probably takes more weight in low power region in thin samples. We further note the oscillation period doesn't depend on pump power, indicating the sound velocity has very weak (if any) dependence on lattice temperature. This also matches the temperature dependent measurement of oscillation period of CLAP (See supporting information).

## Conclusion

In summary, we have observed the CLAP oscillation in multilayer MoS_2_ in time domain. In thinner samples less than 150 L, the generated CLAP shows standing wave nature and the oscillation period is proportional to the sample thickness. In thick samples, the CLAP oscillation period doesn't change with thickness, but proportional to the probe wavelength. From CLAP measurement in time domain, we can calculate the sound velocity in the MoS_2_ to be 7.11*10^3^ m/s. Moreover, both ISBS and laser induced thermo-modulation contribute to the CLAP generation in our experiment, but ISBS is the dominate mechanism in low power excitation region.

### Supporting Information Available

Raman and AFM characterization, additional full scans of transient reflection spectra on sample with different thicknesses, temperature, pump wavelength and polarization dependence of CLAP oscillations.

## Author Contributions

D.S. and J.M.Z. contributed to the conception and design of the experiment; S.F.G. performed the major optical measurement and analysis of the data with assistance of X.F.L., Q.S.W., D.S. and J.M.Z; X.F.Q. and P.H.T. performed the number of layers measurement with Raman and AFM; Z.X. and J.Q. prepared the samples. All authors wrote and reviewed of the manuscript.

## Supplementary Material

Supplementary Information supplementary information
