# Supplementary material for: Coherent Longitudinal Acoustic Phonon Approaching THz Frequency in Multilayer Molybdenum Disulphide
Source: Sci Rep. 2014 Jul 17;4:5722. doi: 10.1038/srep05722 (PMC4101472; doi:10.1038/srep05722)
Supplement: Supplementary Information [file srep05722-s1.docx]

**Supporting information for:**

**Coherent Longitudinal Acoustic Phonon Approaching THz Frequency in Multilayer Molybdenum Disulphide**

Shaofeng Ge^1,2^, Xuefeng Liu^1,2^, Xiaofen Qiao^3^, Qinsheng Wang^1,2^, Zhen Xu^1,2^, Jun Qiu^1,2^, , Pinheng Tan^3^, Jimin Zhao^4^, Dong Sun^1,2,*^

^1^International Center for Quantum Materials, School of Physics, Peking University, Beijing 100871, P. R. China

^2^Collaborative Innovation Center of Quantum Matter, Beijing 100871, P. R. China

^3^State Key Laboratory of Superlattices and Microstructures, Institute of Semiconductors, Chinese Academy of Sciences, Beijing, 100083, P. R. China

^4^Beijing National Laboratory for Condensed Matter Physics and Institute of Physics, Chinese Academy of Sciences, Beijing 100190,P. R. China

*Correspondance and request for materials should be addressed to D. S. (email: [sundong@pku.edu.cn](mailto:sundong@pku.edu.cn))

***Supplementary Materials***

Table of Contents:

S1.Raman and AFM characterization of MoS_2_ samples.

S2.Transient reflection spectra (△R/R) of MoS_2_ film with different thickness.

S3.Temperature dependent measurement of 122L and 1314L samples.

S4.Pump wavelength dependent measurement of 1314 L sample.

S5.Polarization dependent of CLAP oscillations.

S6.Supplementary Reference.

1. **Raman and AFM characterization of MoS_2_ samples**

Low wavenumber Raman measurements are performed using a Jobin-Yvon HR800 system equipped with liquid nitrogen cooled charge-coupled detector. The excitation wavelength is 532 nm from a diode-pumped solid-state laser. Excitation power of 0.23 mW is used to avoid sample heating. The laser plasma lines are removed using a BragGrate bandpass filter (OptiGrate Corp), as these would appear in the same spectral range as the modes of interest. The Rayleigh line is suppressed using four BragGrate notch filters with an optical density 3 and a spectral bandwidth ∼5–10 cm^−1^. The spectral resolution is ∼0.6 cm^−1^, as estimated from the full width at half maximum (FWHM) of the Rayleigh peak.[^1^](#_ENREF_1)

As shown in Fig. S1(a), the 10L sample that is used in the experiment shows the layer breathing (LB) modes (LBMs) peak at 8.9 cm^-1^ and the shear (C) modes peak at 32.3 cm^-1^ , which matches the typical monolayer Raman results in the literature.[^1^](#_ENREF_1)

The AFM measurements were performed using the Dimension Edge system which works at the tapping mode with 0.1 nm resolution in vertical direction and 8~10 nm resolution in transversal direction. Since the thickness of monolayer MoS2 is 0.65 nm[^2^](#_ENREF_2) and the instrumental offset is 1.8 nm, we can get the layer number of the sample from the sample thickness. Figure S1(b) shows the atomic force microscopy (AFM) image of the 124L sample. The height profile clearly showed that the average step height from the substrate to 122L sample is measured to be 85 nm.

| 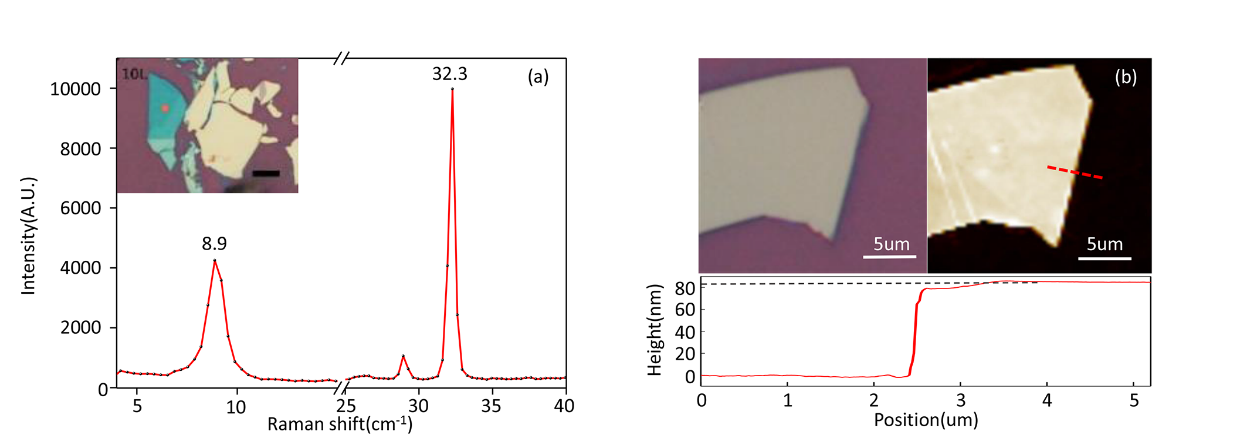  Figure S1: (a) Room temperature Raman spectra of exfoliated 10 layer MoS_2_ sample that’s used in the experiment by 532 nm excitation. The inset shows the micrograph of the sample. (b)The optical and AFM image of the 122L layer sample that’s used in the experiment. The image on the upper left is the optical microscope image of the 122L sample. The upper right is the AFM image and the red dash line indicates the area where the height profile is taken as shown in the bottom. |
| --- |

1. **Transient reflection spectra (DR/R) of MoS_2_ film with different thickness.**

Transient reflection spectra (DR/R) for the MoS_2_ film with different thickness are shown in Figure S2. The pump and probe wavelength are 400 nm and 880 nm respectively with the same pump and probe power. The oscillations are more pronounced with thickness increased. For 1231L and 1314L samples, the oscillations last for several hundred of ps, which are much larger than that of thin samples.

| 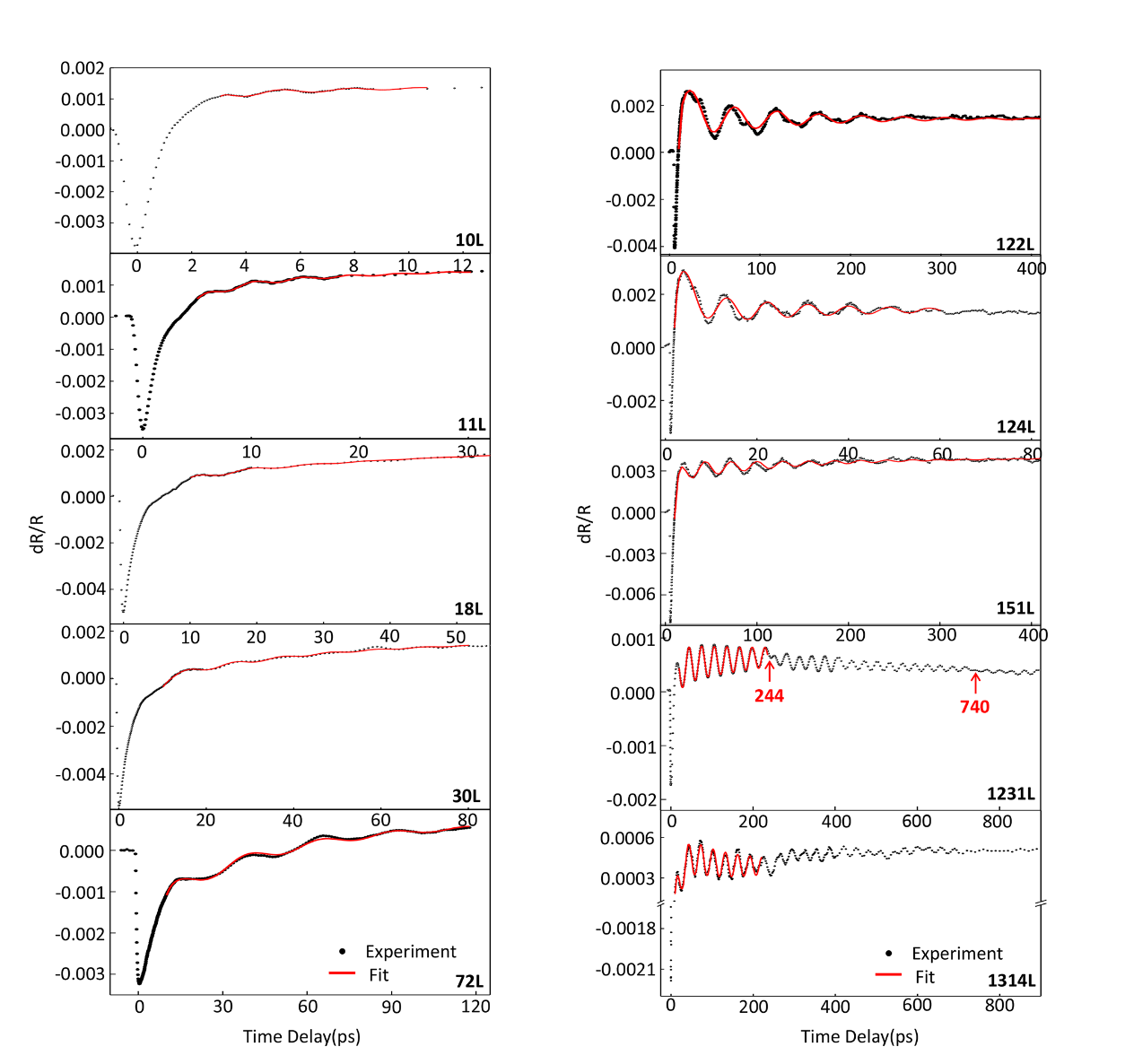  Figure S2: Transient reflection spectra (ΔR/R) of MoS_2_ films with different thicknesses, the number of layers are marked at bottom right. All measurements are performed at room temperature with the same experimental conditions. |
| --- |

1. **Temperature dependent measurement of 72L and 1314L sample.**

In the temperature dependent measurement, the samples are kept in a Janis ST500 cryostat which allows varying the temperature down to 77K with liquid nitrogen. Figure S3 demonstrates the temperature dependent CLAP oscillation of the 72L and 1314L samples. For both samples, the oscillations can be observed at all temperatures from room temperature down to 77 K, and we did not observe temperature dependence of the oscillatory period which means the sound velocity is not sensitive to temperature. On the other hand, the damping time also shows no clear dependence on temperature.

| **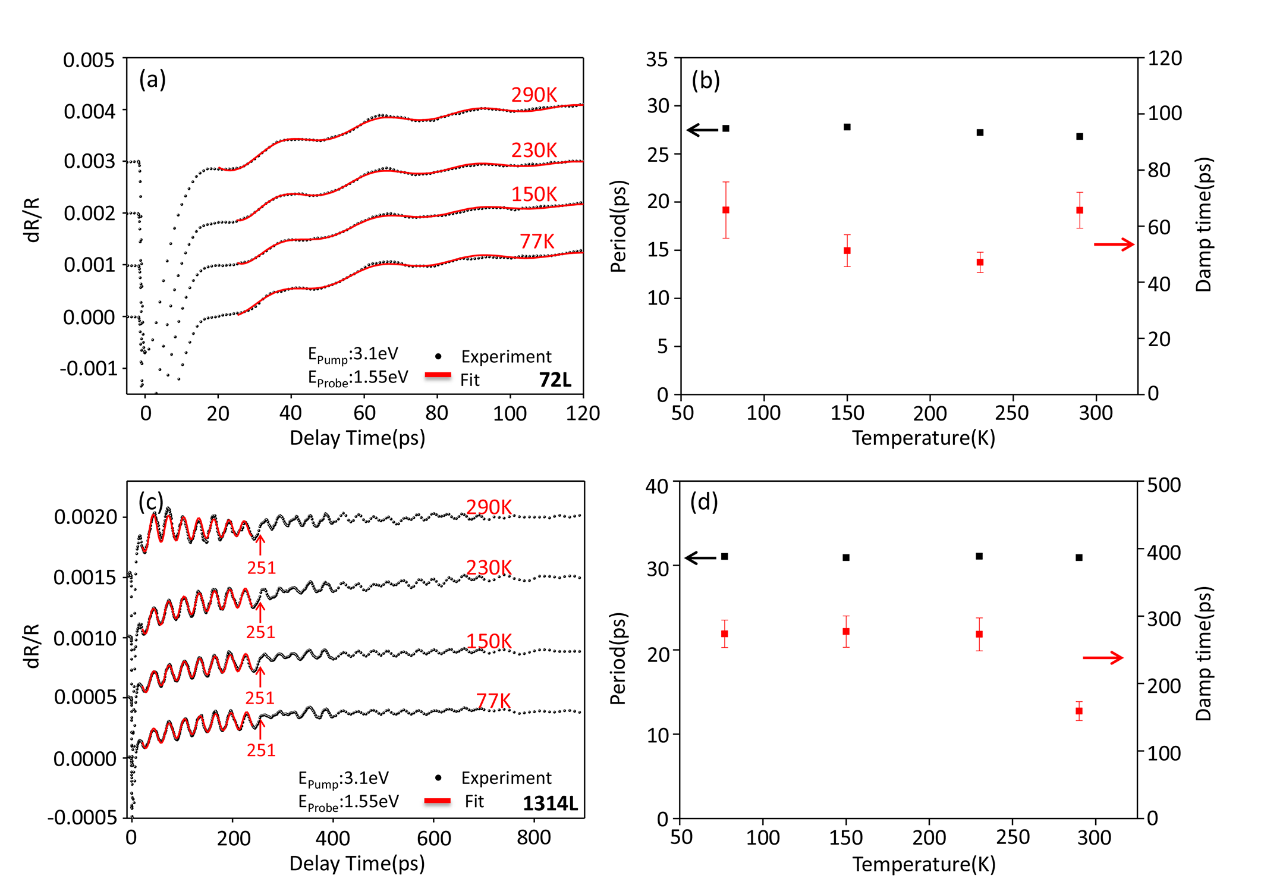**  Figure S3: The temperature dependence of the CLAP oscillations from room temperature down to 77K. (a)(c) The temperature dependence of the CLAP oscillations for the 72L and 1314L samples. The positions of the kinks due to the acoustic echoes are indicated by red arrows in the Figure (c). (b)(d) The temperature dependence of the CLAP oscillation period and damp time for the 72L and 1314L samples. |
| --- |

1. **Pump wavelength dependent measurement of 1314 L sample.**

| **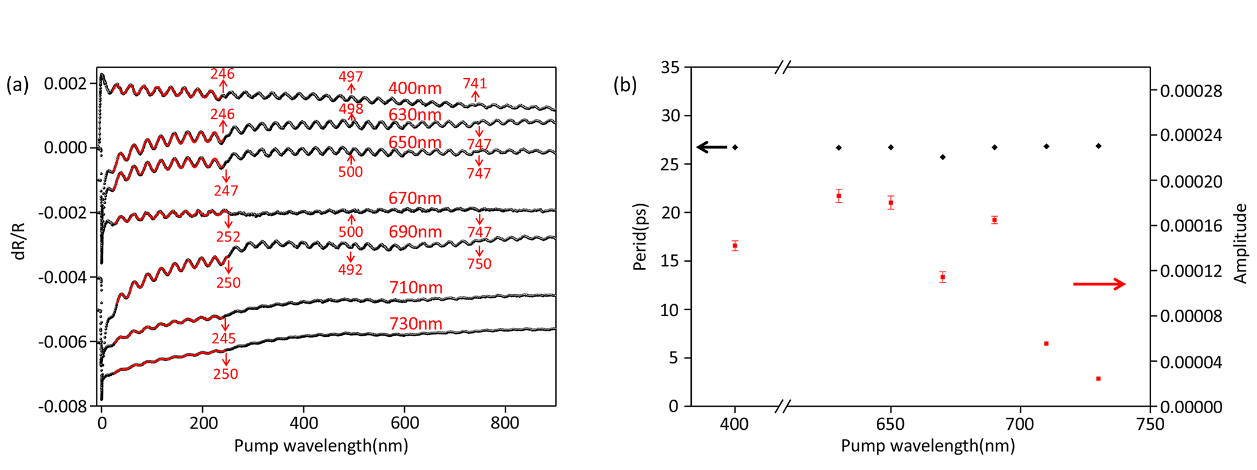**  Figure S4: The pump wavelength dependence of CLAP oscillations at 298K. (a) Transient reflection spectra of 1314L sample with different pump wavelengths (Plots have been shifted vertically for clarity). The positions of the kinks due to the reflected stress pulses are indicated in the figure. (b) The pump wavelength dependence of the oscillation period and amplitude of the coherent phonon oscillations for the 1314L sample. |
| --- |

In the pump wavelength dependent measurements, the probe wavelength was maintained to be 800 nm with pump wavelength varying from 400 nm to 730 nm. As shown in Figure S4, we can see the CLAP oscillation period maintains to be 26.7 ps at different pump wavelength except at 670 nm with oscillation period slightly shifted to 25.7 ps, this probably relates to the A-exciton resonance.[^3^](#_ENREF_3) Meanwhile, the CLAP oscillation amplitude is also relatively smaller when the pump wavelength is 670 nm which is in resonance with the A exciton. The amplitude dependence shows that when the pump wavelength is less than 690nm, the oscillation amplitude is smaller, we speculate this is due to the transition is below the direct gap at K point, although it’s still above the transition of indirect gap.

1. **Polarization dependent of the coherent phonon oscillation**.

The pump and probe beam are tuned to be co- and cross-linear polarized respectively to study the polarization dependence of transient reflection (ΔR). As shown in Figure S5, both CLAP oscillation and carrier dynamics show no polarization dependence on the transient reflection spectra.

| **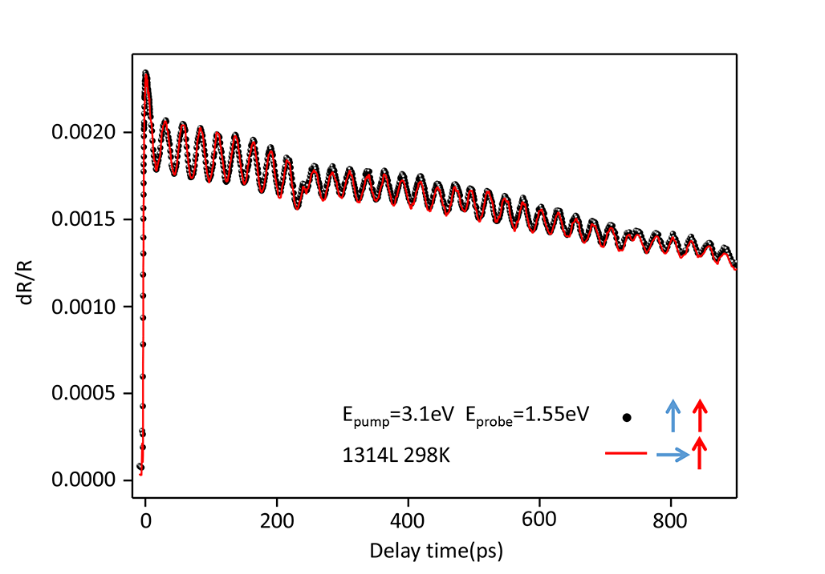**  Figure S5: Transient reflection spectra with co- and cross-linear polarized pump and probe beam on 1314L sample at 298K. |
| --- |

1. **Supplemental references**

1. Zhang, X.; Han, W. P.; Wu, J. B.; Milana, S.; Lu, Y.; Li, Q. Q.; Ferrari, A. C.; Tan, P. H. *Physical Review B* **2013,** 87, (11), 115413.

2. Radisavljevic, B.; Radenovic, A.; Brivio, J.; Giacometti, V.; Kis, A. *Nature nanotechnology* **2011,** 6, (3), 147-150.

3. Beal, A.; Hughes, H. *Journal of Physics C: Solid State Physics* **1979,** 12, (5), 881.
